# Alternative splicing originates different domain structure organization of *Lutzomyia longipalpis* chitinases

**DOI:** 10.1590/0074-02760170179

**Published:** 2018-02

**Authors:** João Ramalho Ortigão-Farias, Tatiana Di-Blasi, Erich Loza Telleria, Ana Carolina Andorinho, Thais Lemos-Silva, Marcelo Ramalho-Ortigão, Antônio Jorge Tempone, Yara Maria Traub-Csekö

**Affiliations:** Fundação Oswaldo Cruz-Fiocruz, Instituto Oswaldo Cruz, Laboratório de Biologia Molecular de Parasitos e Vetores, Rio de Janeiro, RJ, Brasil

**Keywords:** Lutzomyia longipalpis, chitinase, alternative splicing, chitin binding domain

## Abstract

**BACKGROUND:**

The insect chitinase gene family is composed by more than 10 paralogs, which can codify proteins with different domain structures. In *Lutzomyia longipalpis*, the main vector of visceral leishmaniasis in Brazil, a chitinase cDNA from adult female insects was previously characterized. The predicted protein contains one catalytic domain and one chitin-binding domain (CBD). The expression of this gene coincided with the end of blood digestion indicating a putative role in peritrophic matrix degradation.

**OBJECTIVES:**

To determine the occurrence of alternative splicing in chitinases of *L. longipalpis*.

**METHODS:**

We sequenced the *LlChit1* gene from a genomic clone and the three spliced forms obtained by reverse transcription polymerase chain reaction (RT-PCR) using larvae cDNA.

**FINDINGS:**

We showed that *LlChit1* from *L. longipalpis* immature forms undergoes alternative splicing. The spliced form corresponding to the adult cDNA was named LlChit1A and the two larvae specific transcripts were named LlChit1B and LlChit1C. The B and C forms possess stop codons interrupting the translation of the CBD. The A form is present in adult females post blood meal, L4 larvae and pre-pupae, while the other two forms are present only in L4 larvae and disappear just before pupation. Two bands of the expected size were identified by Western blot only in L4 larvae.

**MAIN CONCLUSIONS:**

We show for the first time alternative splicing generating chitinases with different domain structures increasing our understanding on the finely regulated digestion physiology and shedding light on a potential target for controlling *L. longipalpis* larval development.

Leishmaniasis is a serious disease transmitted by sand flies. Transmission occurs when infected females of *Phlebotomus* species in the Old World, and mostly species of the *Lutzomyia* genus in the New World, feed on a vertebrate host (Bates et al. 2015).

As in other hematophagous insects, female sand flies form a peritrophic matrix (PM) around the blood bolus after feeding. The importance of this PM was demonstrated in *Phlebotomus papatasi* flies fed with blood containing an anti-chitinase antibody that led to delay in the onset of egg laying, but longer survival, which might be due to the haeme scavenging capacity of the PM (Robles-Murguia et al. 2014). Also in the Old World vector, when this chitinase gene was silenced there was a decreased infection with *Leishmania major*, indicating the importance of the PM biogenesis for successful parasite development (Coutinho-Abreu et al. 2010). In the New World leishmaniasis vector *Lutzomyia longipalpis* the presence of PM contributes to a favorable environment for the initial survival of *Leishmania* in the sand fly by creating a proteolytic gradient between the midgut lumen and core of the food bolus (Pimenta et al. 1997).

Chitin, which comprises 13% (w/v) of the PM total mass (Peters 1992), is one of the most important biopolymers in nature, being abundant in fungi, arthropods and nematodes (Merzendorfer & Zimoch 2003). In insects this polysaccharide participates in the biogenesis of important structures as the gut and trachea cuticular lining and the exoskeleton (Kramer & Muthukrishnan 1997). Since insect growth and morphogenesis depend on their capacity to remodel chitinous structures, the production of enzymes capable of degrading these polysaccharides is of great importance. Consequently, there is a vast repertoire of chitinases with different structures and functions (Rathore & Gupta 2015).

Insect chitinases belong to the family 18 of glycoside hydrolases (GH18) (Zhu et al. 2008a) which can be divided in eight phylogenetic groups based in their sequence similarity and domain organisation such as presence of linker regions, catalytic and chitin-binding domains (CBD) (Arakane & Muthukrishnan 2010). These groups have distinct physiological roles, which were assigned using expression patterns and RNAi silencing studies in *Drosophila melanogaster* (Zhu et al. 2008b), *Anopheles gambiae* (Zhang et al. 2011) and *Aedes aegypti* (Filho et al. 2002). They are involved in molting, digestion, PM turnover, morphogenesis and cell signaling.

Two chitinases belonging to group IV were described in adult sand flies, one from the New World sand fly *L. longipalpis* (*LlChit1*) (Ramalho-Ortigão & Traub-Cseko 2003) and one from the Old World *P. papatasi* (*PpChit1*) (Ramalho-Ortigão et al. 2005). Both genes are expressed with timing consistent with PM degradation. The chitinase gene here described has high levels of transcription in larvae, as compared to other stages of development, and expression is highest in the larva gut as compared to other tissues (Moraes et al. 2014).

Feeding exogenous chitinase to female *L. longipalpis* can reduce fecundity (Araujo et al. 2012) and chitinase blocking by specific antibody has a deleterious effect on the insect fitness (Robles-Murguia et al. 2014). This shows that chitinases are tuned in order to keep the sand fly balanced physiology.

In this work we present evidence for alternative splicing of the *L. longipalpis LlChit1* gene in sand fly larvae, which originates three spliced forms with different domain structures and the expected bands in Western blot visualised by an anti-chitinase antibody. This is the first time alternative splicing is described in *L. longipalpis* contributing to increased chitinase repertoire.

## MATERIALS AND METHODS

*L. longipalpis rearing and LL5 cell culture* - *L. longipalpis* are routinely maintained in our laboratory insectary. Procedures involving live animals were approved by the FIOCRUZ animal bioethics committee (CEUA - protocol number LW-18/14). *L. longipalpis* embryonic LL5 cells (Tesh & Modi 1983) were grown in L-15 medium (SIGMA) supplemented with 10% fetal bovine serum (Laborclin), 10% tryptose and penicillin/streptomycin (at 100 U/mL and 100 mg/mL, respectively), at 29°C.

*Phylogenetic analysis* - The chitinase sequences of *D. melanogaster* and *A. aegypti* were extracted from GenBank (Zhang et al. 2011). The sequence name, species of origin and identifier are shown in Supplementary data (Table I). *L. longipalpis* putative chitinases annotated from ESTs deposited in GenBank were added to the analysis. Regions corresponding to the catalytic domains from these proteins were selected using the HMMER 3.1 program (hmmer.org) and the HMM model Glyco_ hydro_18 (PF00704). Multiple alignments using the catalytic domain sequences were performed using the ClustalW2 program. The phylogenetic tree was constructed using the MEGA 7.0 program (Kumar et al. 2016). The evolutionary history was inferred by using the Maximum Likelihood method based on the Whelan and Goldman model (Whelan & Goldman 2001) and a bootstrap consensus tree was inferred from 5,000 replicates.

**Fig. 1:**
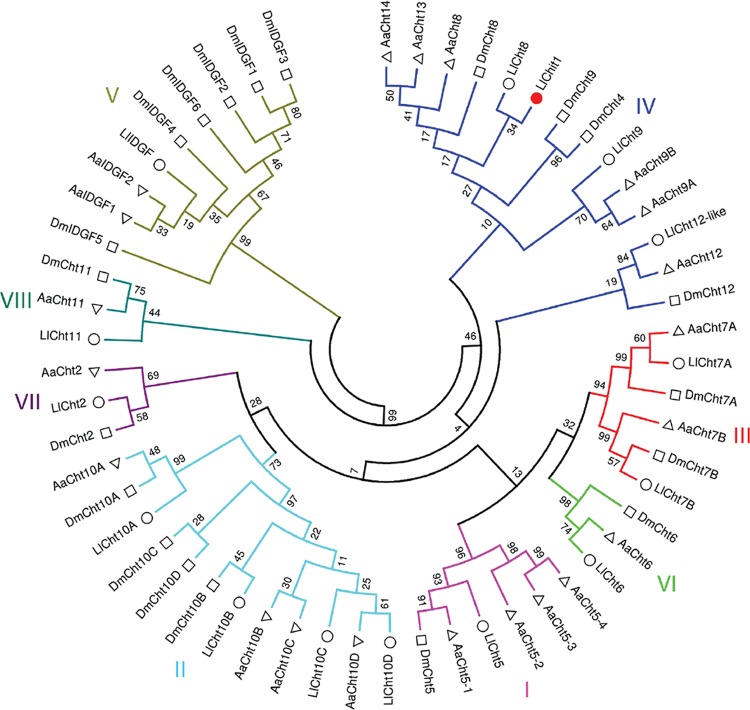
phylogenetic analysis of the chitinase gene family from three different species of dipterans based on the catalytic domain like sequence. Ll: *Lutzomyia longipalpis* (white circles) Aa: *Aedes aegypti* (white triangles); Dm: *Drosophila melanogaster* (white squares). Red circle indicates the *LlChit1* gene. Roman numbers indicate the eight groups of GH18 gene found in different insect genomes. Capital letters after the gene name indicate the corresponding chitinase domain. Numbers on subtree branches indicate bootstrap values.

*Generation and sequencing of a chitinase genomic clone* - DNA was extracted from 10^6^-10^7^ *L. longipalpis* LL5 cells with Illustra Tissue & Cells GenomicPrep Mini Spin Kit (GE Healthcare) and used to produce a genomic library using Lambda EMBL3 phage library kit (Stratagene). The library was plated with XL1-blue cells in soft LB agar plates. Plaques were blotted onto nitrocellulose membranes, the DNA was crosslinked under 120 mJ UV light using Stratalinker UV-Crosslinker (Stratagene) and screened with a 430 bp chitinase fragment obtained from PCR amplification of cDNA as previously described (Ramalho-Ortigão & Traub-Cseko 2003). The probe was randomly labeled with α^32^P-dATP (Amersham Megaprime^TM^ DNA Labeling System, dNTP). Hybridisation was conducted at 45-55°C with the QuickHyb solution (Stratagene). Autoradiography films (X-OMAT XAR-5, Kodak) were exposed to hybridised blots overnight at -70°C. Positive plaques were excised from soft agar plates, diluted and plated for second screening confirmation. The LlChit1 genomic sequence (LlChit1G) was determined by sequencing sub-clones obtained by primer walking using primers described in Supplementary data (Table II, Fig. 1B).

*RNA and cDNA preparation and expression profiling* - *L. longipalpis* larvae, sugar fed males and females, and blood fed females were used for RNA extraction with Trizol (Life Technologies). For cDNA synthesis 5 µg of RNA were treated with 7 U of RQ1 RNase-Free DNase (Promega) and precipitated with ammonium acetate/ethanol. The first complementary strand was synthesised with the use of SuperScript III First-Strand Synthesis System (Invitrogen) and 1 µg of oligo d(T)_12-18_. The transcription profile of *LlChit1* gene was assessed by RT-PCR using specific primers Chit_His_F and Chit_His-R (primers 7 and 12) described in Supplementary data (Table II, Fig. 1C).

*Amplification, cloning and sequencing of the RNA splice forms* - Splice form amplifications were performed with an initial 5 min at 94°C followed by 35 cycles of 45 s at 94°C and 45 s at 55°C, followed by 2 min at 72°C. Forward primers Chit_N-F or Chit_His-F, primers 3 or 7 were used with reverse primer Chit_RNA_2-R, Chit1.2-R or Chit_His-R, primers 6, 8 or 12 indicated in Supplementary data (Table II, Fig. 1D). Amplified fragments were separated in agarose gel, the bands were excised and extraction was performed using the kit Wizard SV Gel and PCR Clean-Up System (Promega). Purified fragments were cloned into pGem-T Easy (Promega) plasmids and clones were sequenced in the PDTIS/FIOCRUZ Sequencing Facility.

*Synthetic peptide and anti-serum production* - An LlChit1 peptide (CEKRQNEKWIDFWDDEQFVPYST) corresponding to the catalytic domain was synthesized by Bio-Synthesis, Inc. (Lewisville, TX, USA) and antibody (α-LlChit1-pep) was produced as described (Telleria et al. 2010). Immunization procedures were approved by the FIOCRUZ animal bioethics committee (CEUA # LW-18/14).

*Western blot* - Different sand fly stages were homogenized in PBS buffer with 0,1 % Triton X-100 and 1 X SigmaFast protease inhibitor cocktail (Sigma-Aldrich-USA), centrifuged at 16.000 x g for 5 min to remove debris. Samples corresponding to 4 insects were separated by SDS-PAGE, transferred to nitrocellulose membrane as previously described (Telleria et al. 2010). After blocking the membrane was incubated for 1 h with α-LlChit1-pep at 1:500 dilution, washed three times for 10 min with TBS and incubated during one hour with HRP-conjugated goat anti-rabbit IgG at a 1:40,000 dilution. The relative molecular mass of the reactive polypeptides was calculated by comparison with the mobility of molecular mass standards, using ImageJ 1.50i software Gel Analysis tools (NIH, USA).

## RESULTS

**Fig. 2:**
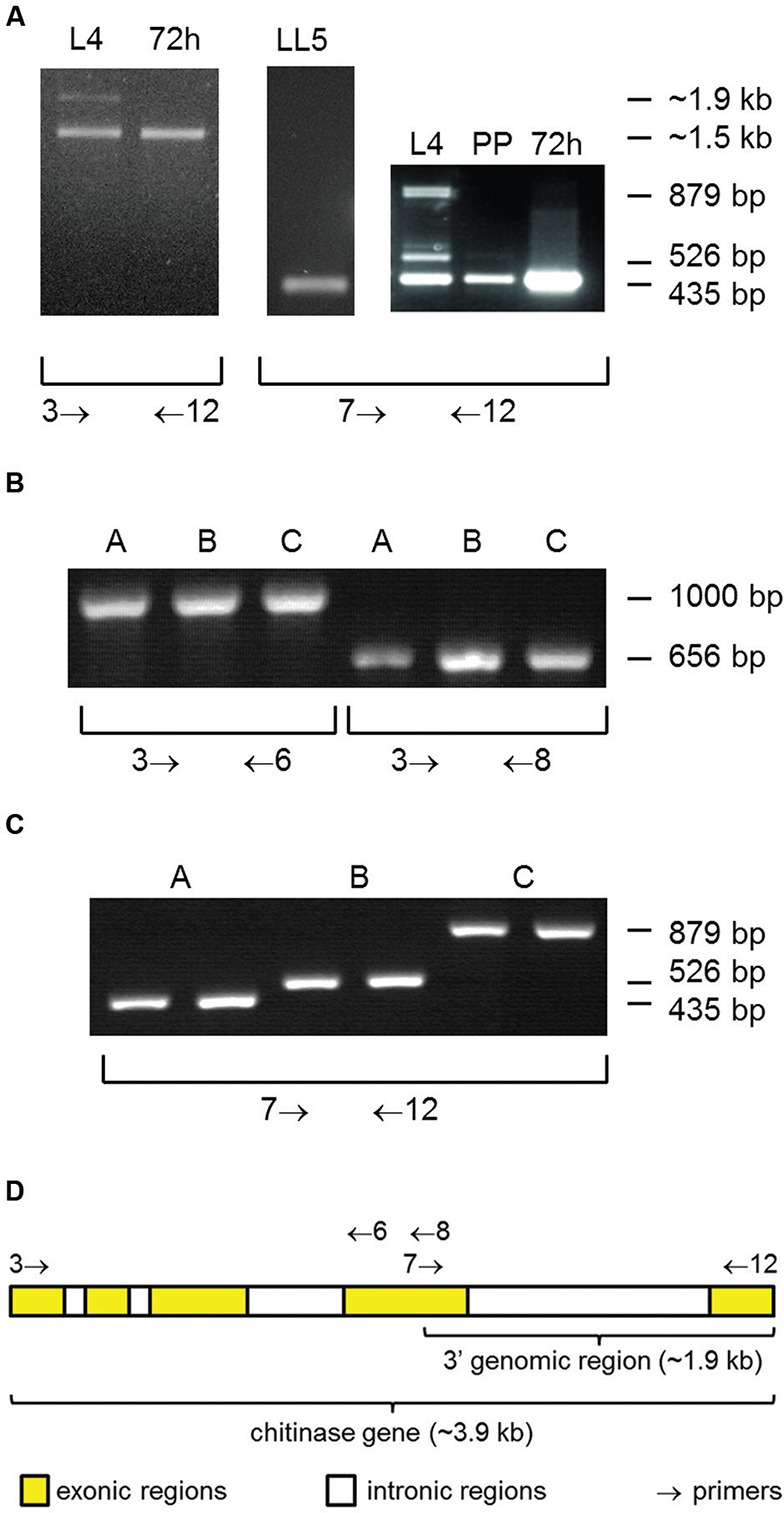
polymerase chain reaction (PCR) amplification of three different spliced forms of *LlChit1* gene. (A) reverse transcription-PCR using RNA extracted from L4 stage and pre-pupae, females 72 h post blood meal and LL5 embryonic cell line. (B) PCR to amply the 5′ extreme of spliced forms A, B and C from the full length cDNA cloned into plasmid vector. It was used one clone of each spliced form per PCR reaction. (C) PCR to amply the 3′ extreme of all three spliced forms from the full length cDNA cloned into plasmid vector. It was used two different clones of each spliced form per primer pair. (D) Schematic representation of oligonucleotides used in PCR and their annealing position on the *LlChit1* gene.

*Determining the genomic sequence of LlChit1* - A genomic clone with more than 10 kb, containing the *LlChit1* gene, was isolated from an *L. longipalpis* genomic library and sequenced by primer walking [Supplementary data (Figs 1A-B, 2)].

*Phylogenetic analysis of L. longipalpis chitinase gene family* - The peptide sequences of the catalytic domain were used to determine the phylogenetic relationship among GH18 gene family of *L. longipalpis*, *D. melanogaster* and *Ae. aegypti* [Supplementary data (Table I), Fig. 1]. The chitinase gene family in the sand fly can be divided into eight groups as seen for other insects. The *LlChit1* gene was grouped among chitinases of group IV that are commonly encoded by several genes in the same species.

*LlChit1 alternative splicing* - To obtain full length mRNA of LlChit1 we performed RT-PCR with RNA extracted from *L. longipalpis* fourth instar (L4) larvae, pre-pupae (PP) or 72 h post blood-feeding (PBF) females and oligonucleotides Chit_N-F and Chit_His-R that anneal on N- and C-terminus regions of the predicted protein [number three and 12; Supplementary data (Table II, Fig. 1C)]. We obtained two products of approximately 1.7 kb and 1.9 kb (not resolved in the gel) amplified from L4 larvae and one product of approximately 1.5 kb from PBF females (Fig. 2A). Additionally, we performed RT-PCR with a forward primer Chit_His-F that anneal on catalytic domain and a reverse primer Chit_His-R that anneals at the end of CBD [primers number 7 and 12; Supplementary data (Table II, Fig. 1C)]. This RT-PCR revealed three PCR products of 435 bp, 526 bp, and 879 bp amplified from L4 larvae and one product of 435 bp amplified from PP and PBF female samples (Fig. 2A). The identified fragments on Fig. 2A were cloned and sequenced, confirming the occurrence of alternative splicing. The transcription of the alternative splicing forms is reduced when L4 larvae stop eating during preparation for pupation at pre-pupae stage (Fig. 2A).

**Fig. 3:**
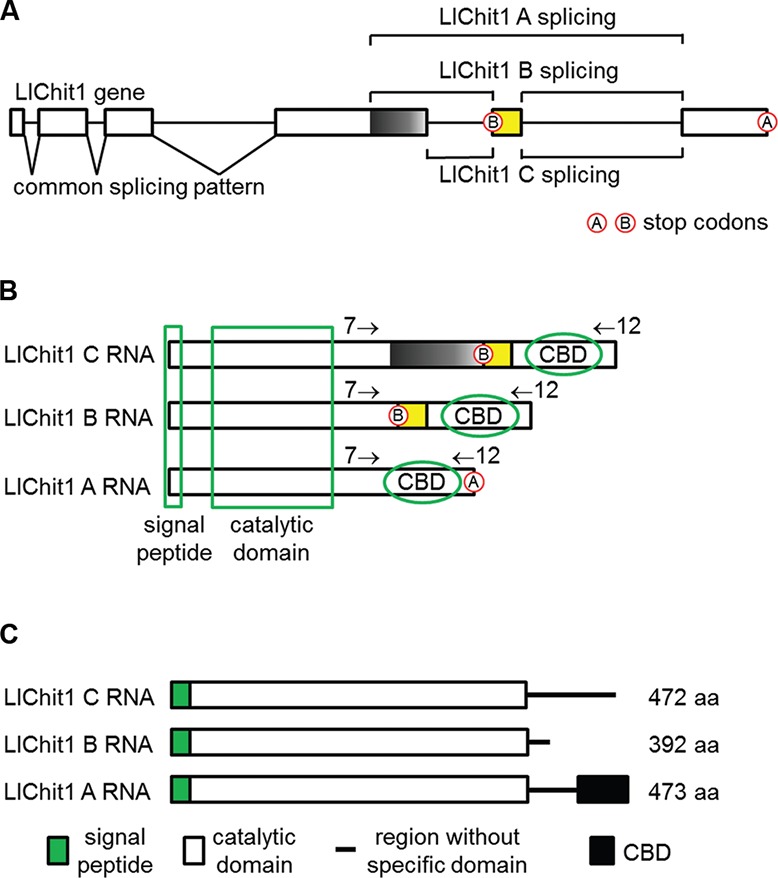
schematic representation of the alternative splicing and its products. (A) *Llchit1* gene and its splicing patterns; lines represent the intron regions; white boxes represent exons present in all spliced forms; yellow box represents the exon present only in spliced forms B and C; black box represents the exon present only in spliced form C. Stop codon A terminates the translation of spliced form A while stop codon B terminates the translation of spliced forms B and C. (B) Representation of transcripts A, B and C. The regions that codify for the signal peptide, catalytic domain and chitin-binding domain (CBD) are market with the green lines. The presence of stop codon B in the spliced forms B and C does not allow the translation of the CBD present at the 3′ end of both spliced forms. (C) Representation of the putative proteins codified by spliced forms A, B and C. All of them possess signal peptide and whole sequence of the catalytic domain, but only form A has a sequence corresponding to CBD.

**Fig. 4:**
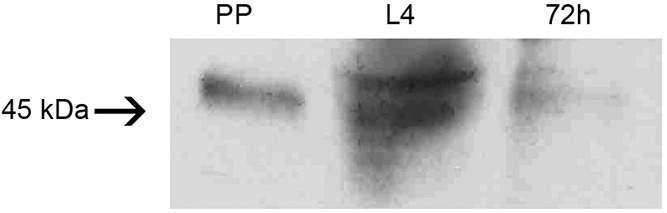
western blot performed with α-LlChit1-pep antibody. Samples corresponding to four insect midguts from PP: pre-pupae; L4: fourth stage larvae; 72 h: female collected at 72 h post blood meal. Standard molecular mass (kDa) is indicated by number and arrow on the left side of the figure.

The splice form previously characterised in adult females was named LlChit1A and the two new splice forms were named LlChit1B and LlChit1C. The sequencing of PCR fragment containing the full length cDNA of approximately 1.5 kb showed that it contained the splice forms A and B [Supplementary data (Figs 3, 4, respectively)] that co-migrated in the agarose gel. The sequencing of PCR fragment containing the full length cDNA of approximately 1.9 kb showed that it contained the splice form C [Supplementary data (Fig. 5)]. After cloning the full length cDNA of the three splice forms we performed PCR experiments with forward primer Chit_N-F and reverse primers Chit_RNA_2-R or Chit1.2-R [primers three, six and eight; Supplementary data (Table II, Fig. 1D)] to confirm that the cloned fragments gave rise to the same amplification pattern seen in the RT-PCR. The amplification of the 5′ side of the three splice forms generated bands with the same size (Fig. 2B) suggesting the existence of a common splicing pattern for introns one, two and three. The PCR using oligonucleotides Chit_His-F and Chit_His-R [primers 7 and 12; Supplementary data (Table II, Fig. 1D)] which anneal to the 3′ extreme of cloned full length cDNAs, amplified the fragments of three different sizes we have obtained previously using RNA extracted from larvae (Fig. 2C).

The annealing sites of oligonucleotides used in the above LlChit1 alternative splicing experiments are represented in Fig. 2D together with graphical representation of exonic and intronic regions of the chitinase gene sequence.

The sequencing of the full length cDNA of the three splice forms [Supplementary data (Fig. 3, 4, 5)] confirmed that the differences among them are observed in the last intron, between the exon that codifies the end of the catalytic domain and the exon that codifies the CBD.

We expressed our overall findings in a graphical representation indicating the common and alternative splicing patterns that occur in the *LlChit1* gene (Fig. 3A). It also shows two intron regions that were identified in RNA sequences and two stop codons located in different positions of the gene that originate the spliced forms (Fig. 3A). Additionally, we represented the LlChit1 spliced forms indicating signal peptide, catalytic domain, CBD regions that were shared among them, introns and stop codons on the RNA sequences (Fig. 3B) or on the predicted amino acid sequences (Fig. 3C).

*Detection of LlChit1 peptide isoforms* - To investigate the translation of LlChit1 alternative spliced mRNAs isoforms we performed Western blot analysis using the α-LlChit1-pep antibody designed against the catalytic domain of the LlChit1 protein, shared by the three isoforms. In PP and 72 h PBF female samples the antibody identified only one band whereas in the L4 larvae sample the same antibody revealed two bands (Fig. 4). The molecular weight range of the observed bands was within the expected for the deduced LlChit1 isoforms predicted by Ex-PASy PeptideMass tool (LlChit1-A: 53.19 kDa, LlChit1-B: 44.05 and LlChit1-C: 53.5 kDa), considering that we did not expect forms A and C to separate in the gel.

## DISCUSSION

Insect chitinases belong to eight groups with different functions. Group I-II chitinases are involved in endocuticle degradation during molting; Group III chitinases are linked to abdominal contraction in early pupation stages and extension of wings; Group IV chitinases are involved in intestinal processes; Group V chitinases may have a role in binding to cell surface receptors (Zhu et al. 2008a).

*L. longipalpis LlChit1* gene is highly transcribed 72 h after blood ingestion and with RNA levels decreasing abruptly in the subsequent 24 h (Ramalho-Ortigão & Traub-Cseko 2003), indicating a role for this chitinase in biogenesis of the PM. We showed that LlChit1 belongs to Group IV chitinases which are expressed only in the gut and participate in the control of the PM thickness and digestion of chitin rich food (Merzendorfer & Zimoch 2003, Arakane & Muthukrishnan 2010).

While gene duplication was one evolutionary process used to produce such variety of chitinase functions (Zhang et al. 2011), alternative gene splicing could well be another. Alternative splicing is very important for insect biology. *D. melanogaster* alternatively processes 31% of its genes (Daines et al. 2011). In *Bombus terrestris* infection with the trypanosomatid *Crithidia bombi* can change the splicing pattern of many genes (Riddell et al. 2014).

The sequencing of a genomic LlChit1 clone with the identification of introns was crucial for the understanding of RT-PCR data that indicated the presence of alternative splicing in *L. longipalpis* larvae. Sequencing of the multiple bands obtained by RT-PCR confirmed the occurrence of alternative splicing. This was confirmed by the band pattern revealed by Western blot using an α-LlChit1-pep antibody. It is expected that the PP and 72 h PBF females will produce only the LlChit1-A isoform and the L4 larvae the three isoforms. The observation of only two peptide bands in the L4 larvae sample is explained by the co-migration of isoforms A and C which have very similar molecular weights (53.19 and 53.5 kDa respectively). The second band represents the LlChit1-B isoform. This outcome is a strong evidence that the *LlChit1* gene is differentially spliced in L4 larvae and that these spliced mRNA isoforms are being translated.

In both larval alternative splicing forms a differential translation process occurs, through the introduction of an alternative stop codon (Fig. 3A-B). Thus the final region of the RNA, which codifies the CBD, gives rise to two putative proteins lacking this domain (Fig. 3C). This type of alternative processing with the introduction of stop codons is widely found in nature. One classical example in insects is found in *D. melanogaster* where the introduction of a stop codon in an initial exon of a gene coding for a protein determinant for female sexual differentiation leads to loss of function and generation of a male phenotype (Schutt & Nothiger 2000).

The putative proteins produced by the alternatively processed LlChit1 RNAs possess a complete catalytic domain and possibly catalytic activity. Nevertheless, the loss of the CBD must reduce the protein affinity for the substrate, as already reported for chitinases with the same architecture (Zhu et al. 2008b). Alternative processing of insect chitinase genes has already been described, but in all cases the domain architecture has been preserved. A chitinase gene of *Bombyx mori* originates four different transcripts with catalytic domain and CBD, showing catalytic activity (Abdel-Banat & Koga 2002).

As previously reported, the putative product of LlChit1A possessing a catalytic domain and a CBD is present in the adult female *L*. *longipalpis* gut 48-72 h post-blood meal (Ramalho-Ortigão & Traub-Cseko 2003), when the PM is being degraded. This suggests a role for this chitinase form in the breaking of PM chitin fibrils. Although chitin is a very important component of the PM, it is not the main component of the matrix, being in close contact with various proteins containing CBDs and also with glycoproteins, making the access of chitinase to its substrate quite difficult. This requires the existence of a CBD for the efficient hydrolytic functions, determinant for successful degradation.

Immature forms of sand flies feed on organic decomposing matter, which includes fungi. These have a chitin rich cell wall, being able to furnish high amounts of N-acetyl-glucosamine for the growing larvae. At the same time, producing digestive enzymes that can digest food chitin without harming the insect PM, which is fundamental in the compartmentalisation of digestion, would require a finely specialised chitinase repertoire.

PM can be classified in two types, type 1 PM that is formed and degraded according to female blood digestion, or type 2 PM that is constantly produced and degraded in the larval gut (Lehane 1997, Secundino et al. 2005). Although they differ in biogenesis pattern, their protein composition is similar (Tellam et al. 1999, Hegedus et al. 2009). Since chitinase transcript LlChit1A containing the CBD is present in both larvae and adult *L. longipalpis*, where PM formation and degradation are required, it is probably involved in PM chitin digestion and therefore PM biogenesis regulation. LlChit1B and LlChit1C transcripts without the CBD most probably have a role in digesting exogenous chitin ingested by larvae. The investigation of the different chitinase splice forms activity is of great interest and included in our future plans.

If in other insects the presence of paralogous chitinase genes with or without the CBD has been reported (Zhu et al. 2008a), *L. longipalpis* seems to be handling the chitinase diversity challenge also by using alternative splicing.
