# Supplementary material for: Alternative splicing originates different domain structure organization of Lutzomyia longipalpis chitinases
Source: Mem Inst Oswaldo Cruz. 2018 Feb;113(2):96–101. doi: 10.1590/0074-02760170179 (PMC5722264; doi:10.1590/0074-02760170179)
Supplement: Supplementary file 1 [file 0074-0276-mioc-113-02-0096-Suppl01.pdf]

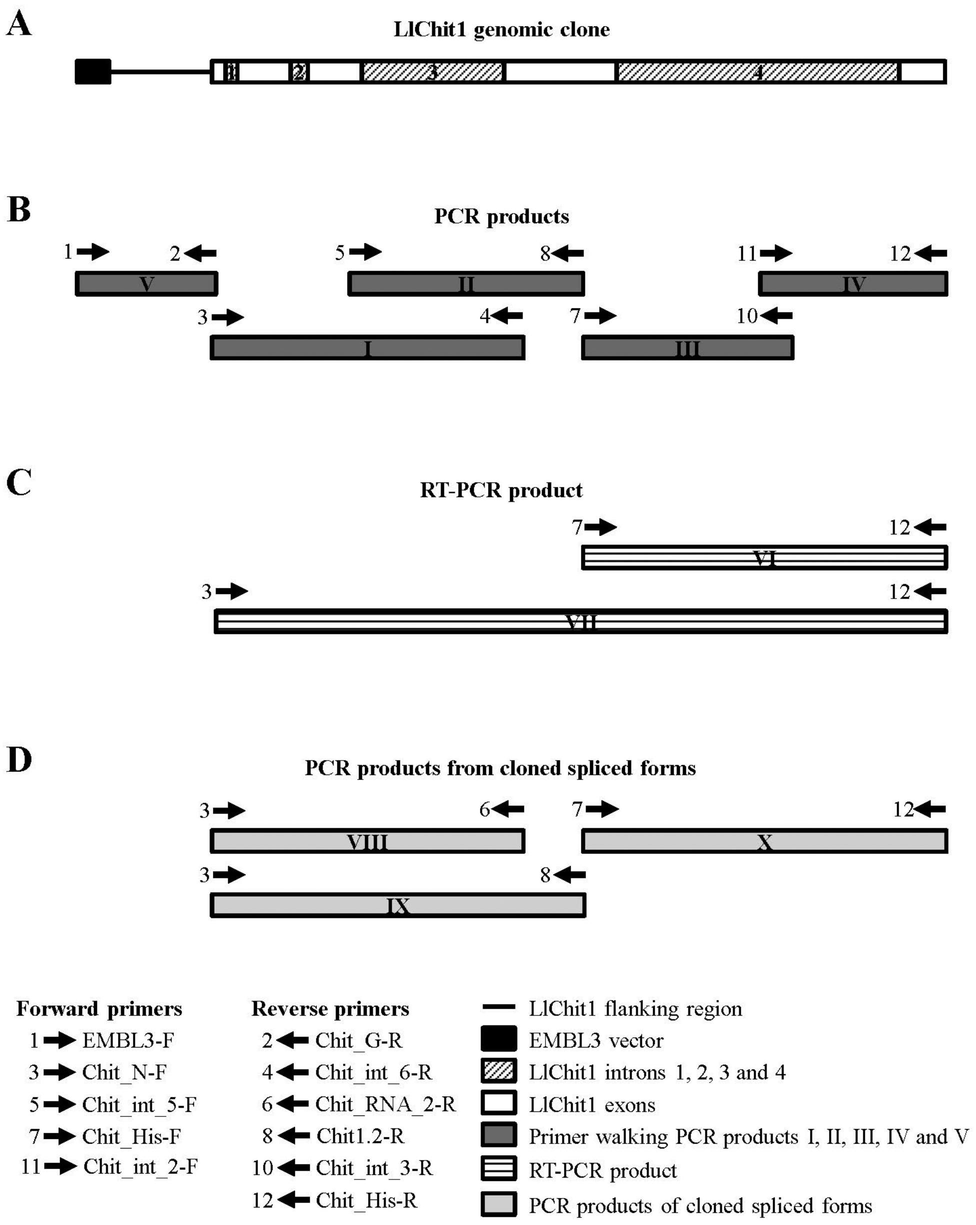

Fig. 1: primers, polymerase chain reaction (PCR) for LlChit1 sequence confirmation and reverse transcription-PCR (RT-PCR): (A) LlChit1 genomic clone is represented by rectangles and line: black rectangle represents a segment of EMBL3 vector, black line represents LlChit1 flanking region, white rectangles represent LlChit1 exons, textured rectangles 1, 2, 3 and 4 represent LlChit1 introns. PCR and RT-PCR products are represented below genomic clone aligned with the corresponding primer annealing sites. (B) Dark grey rectangles I, II, III, IV and V represent PCR products amplified for LlChit1 sequencing confirmation. (C) Textured rectangle VI and VII represent LlChit1A RT-PCR products. (D) Light grey rectangles VIII, IX and X represent PCR products from cloned spliced forms. Arrows and numbers represent primer used for sequencing, PCR and RT-PCR also described on Supplementary data (Table II).

LlChit1 genomic sequence

```
   1  atgaagacgttggtgtttctgtgtgtcgccttatcaattttgggcttggcagtaactgaa
       M  K  T  L  V  F  L  C  V  A  L  S  I  L  G  L  A  V  T *E    20
  61  aGTgagagtgttttttttcttcgtgtgatatataattttgtgacgcaaattcttcattt
 121  ttttacgtatAGaaaaaattgtctgctatcacggtacgtggtcctactaccggcagggaa
                   K  K  I  V  C  Y  H  G  T  W  S  Y  Y  R  Q  G    36
 181  atggaaaattcggagtagcacaaattgatccattcctctgtacgcatttggtttatacgt
      N  G  K  F  G  V  A  Q  I  D  P  F  L  C  T  H  L  V  Y  T     56
 241  ttttcgggatttcttccgagggtgggattagaattctcgatccttacttggatctcgatg
      F  F  G  I  S  S  D  G  G  I  R  N  L  D  P  Y  L  D  L  D     76
 301  agaactacggccttgggaatataagaaaattcaatgaactcaagaaagttaatcccaaat
      E  N  Y  G  L  G  N  I  R  K  F  N  E  L  K  K  V  N  P  K     96
 361  tgaagacaatcgccggtgttggaggatggaatgaaggaagtgttacattttctcagGTca
      L  K  T  I  A  G  V  G  G  W  N  E  G  S  V  T  F  S  Q       115
 421  gtgaaacagatctcacgattttaagaagatttcatttgagactttttcctctattAGg
 481  tcgtcaatgatccacgcaagaggcagaatttcgtgaagaattcgttggaattttgaaaa
      V  V  N  D  P  R  K  R  Q  N  F  V  K  N  S  L  E  F  L  K    135
 541  aatacaattttaatggtcttgacgttgactgggagtatccagcacaacgtggagggaatc
      K  Y  N  F  N  G  L  D  V  D  W  E  Y  P  A  Q  R  G  G  N    155
 601  aggataaggataaggaagcctacactctcctcctcaaggaactctcggaattcctacatc
      Q  D  K  D  K  E  A  Y  T  L  L  L  K  E  L  S  E  F  L  H    175
 661  ctaaaggctacagcctctccgctgctgtagcatcagcggaattttcagctaagatctctt
      P  K  G  Y  S  L  S  A  A  V  A  S  A  E  F  S  A  K  I  S    195
 721  acaacatcgctgaggtttcaaaGTaagttcaaaggaatttttagggaagattgggctaa
      Y  N  I  A  E  V  S  K                                         203
 781  agctattcaaatgagtatgaaaaaatggaaaattaattaaaatttgctttaaaaacattt
 841  tttgcaaggtttgagaacagtttttgttcaaaaatttaagatctaaagaccgtaaaaaat
 901  caaaatcccaataattaataaatttgggaaagtaatttattcataaaaacttaattttac
 961  actcaaagctttcttaaataattcattttttcccttaattttttttccaacttcaaacat
1021  tgaaaaggacgcgttcgcaatttttcacaaagatttgtttccacaaattttaaatttc
1081  cggttatattgaaagctaaaattatcaatactaaaacttacttttcctatgtgttttgag
1141  agttaataggtcaggcacaaaaactcttcactgataatgaaaattccctttaatccaatg
1201  ccaacaatgctaagtcaagtggtcaccatcttattgaacttgacacaaatttaaatcaaa
1261  tattacacaaaggggggcgctaattgtaccaaagcgcaaatggattgaattttaaaaagaa
1321  acgaataattataacgcccaaagtacaagaacagaaactttctcaagaatttcccgaatt
1381  tccttttgaaaatcaatttttttgtgctgcaaatacttccagctcttcttatctttcctca
1441  agttcaagattaatatcaagagtctttggggattgtcttgtctgcaaaatatgactgaag
1501  cacctctcttgcAGatatctggatttcattggggtaatgacgtatgatttacacggatca
                      Y  L  D  F  I  G  V  M  T  Y  D  L  H  G  S    218
1561  tgggatccaaagattgggaataatgcgccattgtatgcaggatcgtgggaacaaacggag
       W  D  P  K  I  G  N  N  A  P  L  Y  A  G  S  W  E  Q  T  E   238
1621  cttgagaagcaattaaatgttgacgccgctataaagtactggctcagcaatggaggtgcc
       L  E  K  Q  L  N  V  D  A  A  I  K  Y  W  L  S  N  G  G  A   258
1681  cctgagaagctcctcctgggcgttccactgtacggtcgtggcttccgcatggttaatggt
       P  E  K  L  L  L  G  V  P  L  Y  G  R  G  F  R  M  V  N  G   278
1741  cagagtaaaccaggatccgttcacggagggccttgtcaggctgggccctacactcaaact
       Q  S  K  P  G  S  V  H  G  G  P  C  Q  A  G  P  Y  T  Q  T   298
1801  cccggcatgatgggcttcaatgagctctgcgagaagcgtcggaatgaaaaatggattgat
       P  G  M  M  G  F  N  E  L  C  E  K  R  R  N  E  K  W  I  D   318
1861  ttctgggatgatgagcaatttgttccgtactccacaaagaacgatcagtggattggattt
       F  W  D  D  E  Q  F  V  P  Y  S  T  K  N  D  Q  W  I  G  F   338
1921  gatgatgagaagagtataaaattcaaatcaaactacgtcaacagccacaatcttggtggg
       D  D  E  K  S  I  K  F  K  S  N  Y  V  N  S  H  N  L  G  G   358
1981  gtgattgtgtggtccattgaaacggatgattttagaggattctgcggaagaggaactttc
       V  I  V  W  S  I  E  T  D  D  F  R  G  F  C  G  R  G  T  F   378
2041  ccactactcaaggagctcaatgcatccctccttggtaacaatGTgagtacctccaatgaa
       P  L  L  K  E  L  N  A  S  L  L  G  N  N                     392
2101  acagatcccgagatcatgtccctcctttccgcggagaggaagaaaacagcagcattgaag
2161  aaattcattcatgatttcttcttcaagaccttccagccaaagtatcagaaactcattcag
2221  ttcaatttactcaaagaaaaatatttttccgaaagcttcaccgaactctacggtacatac
2281  gttcaaatgctctcagacacatacaagagtgaaaatgatttataagcaggaattaagttt
2341  atttttctatacttaaccctttagcgtccaacgtgaaacactaaaacattgaaacatgc
2401  gctcttttatcgcgatttttattctatgaatttttttagaggacttttctcaatcagaac
2461  gtcttctttgaccccttatgcgtgaattggatgcatttgtaacatggaaaaacaattaca
2521  ttcttaaataattgaaaaaataaaatgtttcacgtttgggtcagcgtatgacccaaaaaa
2581  cgttaaaagggttaattttgtgttttttttttattgagaaataagtatttatagtctct
2641  aatggattaatggtcataaatatacacttacttaatgcaatttacggaaaataaaatcac
2701  tttgcgcggatttcaagtgatttagcacttgagggcattcttcattggctgaaagggtgc
2761  aagctttgtttttttgctgcacttccggatatctacgaaagcaggaagagttaagtagag
2821  ttcaatttatcaatgcaaaaacaatatttaacgtaaattgcatgttttgcaactaatcgc
2881  atgacttataacaatcgctaaatcccctaaatggttctttttttaagatttaaataaaaa
2941  acttcgagctttgaaaacgcgagcataattgttaactgttgaaaagtttctctttaagct
3001  tttataagctgctttacacaacgctttatacctaaactgcaataaactctgagcctactg
3061  tcaaatcgggttactttaaatatgaggggtcgattctctccaaaaatttcttttattct
3121  tttcttgtagactcgaaagggttaaaatgcacaaaacattcacactaatgctttgcgatg
3181  atttttttcacaaataatataattttatgcttcatccattaaaagagacaaaaaataca
3241  acagtgacgtcattgcttgtatttttaagcaaatatttgccggcttttgcatgctgattt
3301  ctagtagaaaaatttggggagtttcttaggcaatttctaatacttaatcaatcacatttt
3361  tttctatattgttctttcaaagattttgctcgaatcagaagttccccaatacaaaaatga
3421  ataaacttctttaatttaacgtttgaacacgaatcaaagaatctcattgtacaaaatttc
3481  acaatttatttattcttacaattaatttttgtattaaattaacatttaatccttttggtg
3541  ttgtAGcacacctggacacctccatcaacatcaaccaccgaatggaatcccaatccaacg
             H  T  W  T  P  P  S  T  S  T  T  E  W  N  P  N  P  T   410
3601  tccacaacgaaacctccaccaatcctcccaagtcccgaatgtaccgaagatggttacttt
       S  T  T  K  P  P  P  I  L  P  S  P  E (C) T  E  D  G  Y  F   430
3661  agggatccaaatgactgcagtatcttccaccaatgcatcaatgggactcaatataccttc
       R  D  P  N  D (C) S  I  F  H  Q (C) I  N  G  T  Q  Y  T  F   450
3721  ttctgcccacatggtcttgtcttcgatccagccatcatagcttgcaattggccccatata
       F (C) P  H  G  L  V  F  D  P  A  I  I  A (C) N  W  P  H  I   470
3781  gtgcaatgctga
       V  Q (C) -                                                   473
```

Fig. 2: LlChit1 genomic sequence: numbers on left: nucleotide position. On the right: amino acid positions. Asterisk: putative signal peptide cleavage site. Underlined lower case letters: introns. Underlined upper case bold letters: nucleotides possibly involved in splicing. Underlined upper case red letters: chitin binding domain. Letters in parenthesis: conserved cysteine residues. Red letters: conserved catalytic domain. Red underlined bold letters: catalytic core.

Supplementary data

LlChit1A

```
   1 atgaagacgttggtgtttctgtgtgtcgccttatcaattttgggcttggcagtaactgaa
      M  K  T  L  V  F  L  C  V  A  L  S  I  L  G  L  A  V  T* E   20
  61 aaaaaaattgtctgctatcacggtacgtggtcctactaccggcagggaaatggaaaattc
      K  K  I  V  C  Y  H  G  T  W  S  Y  Y  R  Q  G  N  G  K  F   40
 121 ggagtagcacaaattgatccattcctctgcacgcatttggtttatacgtttttcgggatt
      G  V  A  Q  I  D  P  F  L  C  T  H  L  V  Y  T  F  F  G  I   60
 181 tcttccgagggtgggattagaattctcgatccttacttggatctcgatgagaactacggc
      S  S  E  G  G  I  R  I  L  D  P  Y  L  D  L  D  E  N  Y  G   80
 241 cttgggaatataagaaaattcaatgaactcaagaaagttaatccaaaattgaagacaatc
      L  G  N  I  R  K  F  N  E  L  K  K  V  N  P  K  L  K  T  I  100
 301 gccggtgttggaggatggaatgaaggaagtgttacatttctcaggtcgtcaatgatcca
      A  G  V  G  G  W  N  E  G  S  V  T  F  S  Q  V  V  N  D  P  120
 361 cgcaagaggcagaatttcgtgaagaattcgttggaattttgaaaaaatacaattttaat
      R  K  R  Q  N  F  V  K  N  S  L  E  F  L  K  K  Y  N  F  N  140
 421 ggtcttgacgttgactgggagtatccagcacaacgtggagggaatcaggaaaaggataaa
      G  L  D  V  D  W  E  Y  P  A  Q  R  G  G  N  Q  E  K  D  K  160
 481 gaagcctacacgctcctcctcaaggaactctcggaattcctacatcctaaaggctacact
      E  A  Y  T  L  L  L  K  E  L  S  E  F  L  H  P  K  G  Y  T  180
 541 ctctccgctgctgtagcatcagcggaattttcagctaaaatctcttacaacatcgctgag
      L  S  A  A  V  A  S  A  E  F  S  A  K  I  S  Y  N  I  A  E  200
 601 gtttcaaaatatctggatttcattggggtaatgacgtatgatttacacggatcatgggat
      V  S  K  Y  L  D  F  I  G  V  M  T  Y  D  L  H  G  S  W  D  220
 661 ccaaagattgggaataatgcgcctttgtatgcgggatcgtgggagcaaacggagcttgag
      P  K  I  G  N  N  A  P  L  Y  A  G  S  W  E  Q  T  E  L  E  240
 721 aagcaattaaatgttgacgctgctataaagtactggctcagcaatagcggggctcctgag
      K  Q  L  N  V  D  A  A  I  K  Y  W  L  S  N  S  G  A  P  E  260
 781 aagctcctcctgggcgttccactgtacggtcgtggcttccgcatggttaatggtcagagt
      K  L  L  L  G  V  P  L  Y  G  R  G  F  R  M  V  N  G  Q  S  280
 841 aaaccaggatccgttcatggaggtccttgtcaggctgggccctacactcaaactcccggc
      K  P  G  S  V  H  G  G  P  C  Q  A  G  P  Y  T  Q  T  P  G  300
 901 atgatgggcttcaatgagctctgcgagaagcgtcagaatgaaaaatggattgatttctgg
      M  M  G  F  N  E  L  C  E  K  R  Q  N  E  K  W  I  D  F  W  320
 961 gatgatgagcaatttgttccctactccacaaagaacgatcagtggattggatttgatgat
      D  D  E  Q  F  V  P  Y  S  T  K  N  D  Q  W  I  G  F  D  D  340
1021 gagaagagtataaaattcaaatcaaattacgtcaacagccacaatcttggtggtgtgatt
      E  K  S  I  K  F  K  S  N  Y  V  N  S  H  N  L  G  G  V  I  360
1081 gtgtggtccattgaaacagatgattttagaggattctgcggaagaggaaccttcccactt
      V  W  S  I  E  T  D  D  F  R  G  F  C  G  R  G  T  F  P  L  380
1141 ctcaaggagctcaatgcatccctccttggtaacaatcacacctggacacctccatcaaca
      L  K  E  L  N  A  S  L  L  G  N  N  H  T  W  T  P  P  S  T  400
1201 tcaaccaccgaatggaatcccaatccaacgtccacaacgaaacctccaccaatcctccca
      S  T  T  E  W  N  P  N  P  T  S  T  T  K  P  P  P  I  L  P  420
1261 agtcccgaatgtaccgaagatggttactttagggatccaaatgactgcagtatcttccac
      S  P  E (C) T  E  D  G  Y  F  R  D  P  N  D (C) S  I  F  H  440
1321 caatgcatcaatgggactcaatatacattcttctgcccacatggtcttgtcttcgatcca
      Q (C) I  N  G  T  Q  Y  T  F  F (C) P  H  G  L  V  F  D  P  460
1381 gccatcatagcttgcaattggccccatatagtgcaatgctga
      A  I  I  A (C) N  W  P  H  I  V  Q (C) -                     473
```

Fig. 3: LlChit1A spliced form sequence: numbers on left side: nucleotide position. Numbers on right: amino acid position. Asterisk: putative signal peptide cleavage site. Underlined letters: chitin binding domain. Letters in parenthesis: conserved cysteine residues. Red letters: conserved catalytic domain. Red underlined bold letters: catalytic core.

LlChit1B

```
1     atgaagacgttggtgtttctgtgtgtcgccttatcaattttgggcttggcagtaactgaa
       M  K  T  L  V  F  L  C  V  A  L  S  I  L  G  L  A  V  T* E      20
61    aaaaaaattgtctgctatcacggtacgtggtcctactaccggcagggaaatggaaaattc
       K  K  I  V  C  Y  H  G  T  W  S  Y  Y  R  Q  G  N  G  K  F      40
121   ggagtagcacaaattgatccctttctctgcacgcatttggtttatacgtttttcgggatt
       G  V  A  Q  I  D  P  F  L  C  T  H  L  V  Y  T  F  F  G  I      60
181   tcttccgagggtgggattagaattctcgatccttacttggatctcgatgagaactacggc
       S  S  E  G  G  I  R  I  L  D  P  Y  L  D  L  D  E  N  Y  G      80
241   cttgggaatataagaaaattcaatgaactcaagaaagttaatccaaaattgaagacaatc
       L  G  N  I  R  K  F  N  E  L  K  K  V  N  P  K  L  K  T  I      100
301   gccggtgttggaggatggaatgaaggaagtgttacattttctcaggtcgtcagtgatcca
       A  G  V  G  G  W  N  E  G  S  V  T  F  S  Q  V  V  S  D  P      120
361   cgcaagaggcagaatttcgtgaagaattcgttggaattttttgaaaaaatacaattttaat
       R  K  R  Q  N  F  V  K  N  S  L  E  F  L  K  K  Y  N  F  N      140
421   ggtcttgacgttgactgggagtatccagcacaacgtggagggaatcaggataaggataaa
       G  L  D  V  D  W  E  Y  P  A  Q  R  G  G  N  Q  D  K  D  K      160
481   gaagcctacacgctccttctcaaggaactctcggaattcctacatcctaaaggctacact
       E  A  Y  T  L  L  L  K  E  L  S  E  F  L  H  P  K  G  Y  T      180
541   ctctccgctgctgtagcatcagcggaattttcagctaaaatctcttacaacatcgctgag
       L  S  A  A  V  A  S  A  E  F  S  A  K  I  S  Y  N  I  A  E      200
601   gtttcaaaatatctggatttcattggggtaatgacgtatgatttacacggatcatgggat
       V  S  K  Y  L  D  F  I  G  V  M  T  Y  D  L  H  G  S  W  D      220
661   ccaaagattgggaataatgcgcctttgtatgcgggatcgtgggagcaaacggagcttgag
       P  K  I  G  N  N  A  P  L  Y  A  G  S  W  E  Q  T  E  L  E      240
721   aagcaattaaatgttgacgctgctataaagtactggctcagcaatagcggggctcctgag
       K  Q  L  N  V  D  A  A  I  K  Y  W  L  S  N  S  G  A  P  E      260
781   aagctcctcctgggcgttccactgtacggtcgtggcttccgcatggttaatggtcagagt
       K  L  L  L  G  V  P  L  Y  G  R  G  F  R  M  V  N  G  Q  S      280
841   aaaccaggatccgttcatggaggtccttgtcaggctgggccctacactcaaactcccggc
       K  P  G  S  V  H  G  G  P  C  Q  A  G  P  Y  T  Q  T  P  G      300
901   atgatgggcttcaatgagctctgcgagaagcgtcagaatgaaaaatggattgatttctgg
       M  M  G  F  N  E  L  C  E  K  R  Q  N  E  K  W  I  D  F  W      320
961   gatgatgagcaatttgttccctactccacaaagaacgatcagtggattggatttgatgat
       D  D  E  Q  F  V  P  Y  S  T  K  N  D  Q  W  I  G  F  D  D      340
1021  gagaagagtataaaattcaaatcaaattacgtcaacagccacaatcttggtggggtgatt
       E  K  S  I  K  F  K  S  N  Y  V  N  S  H  N  L  G  G  V  I      360
1081  gtgtggtccattgaaacagatgattttagaggattctgcggaagaggaaccttcccactt
       V  W  S  I  E  T  D  D  F  R  G  F  C  G  R  G  T  F  P  L      380
1141  ctcaaggagctcaatgcatccctccttggtaacaattgatttagcacttgagggcattct
       L  K  E  L  N  A  S  L  L  G  N  N  -                           392
1201  tcattggctgagagggtgcgagctttgttttctgctgcacttccggatatctacgaaagc
1261  aggaagacacacctggacacctccatcaacatcaaccaccgaatggaatcccaatccaac
1321  gtccacaacgaaacctccaccaatcttcccaagtcccgaatgtaccgaagatggttactt
1381  tagggatccaaatgactgcagtatcttccaccaatgcatcaatgggactcaatatacatt
1441  cttctgcccacatggtcttgtcttcgatccagccatcatagcttgcaattggccccatat
1501  agtgcaatgctga
```

Fig. 4: LlChit1B spliced form sequence: numbers on left side: nucleotide position. Numbers on right side: amino acid position. Asterisk: putative signal peptide cleavage site. Red letters: conserved catalytic domain. Red underlined bold letters: catalytic core.

LlChit1C

```
   1 atgaagacgttggtgtttctgtgtgtcgccttatcaattttgggcttggcagtaactgaa
      M  K  T  L  V  F  L  C  V  A  L  S  I  L  G  L  A  V  T* E    20
  61 aaaaaaattgtctgctatcacggtacgtggtcctactaccggcagggaaatggaaaattc
      K  K  I  V  C  Y  H  G  T  W  S  Y  Y  R  Q  G  N  G  K  F    40
 121 ggagtagcacaaattgatccctttctctgcacgcatttggtttatacgtttttcgggatt
      G  V  A  Q  I  D  P  F  L  C  T  H  L  V  Y  T  F  F  G  I    60
 181 tcttccgagggtgggattagaattctcgatccttacttggatctcgatgagaactacggc
      S  S  E  G  G  I  R  I  L  D  P  Y  L  D  L  D  E  N  Y  G    80
 241 cttgggaatataagaaaattcaatgaactcaagaaagttaatccaaaattgaagacaatc
      L  G  N  I  R  K  F  N  E  L  K  K  V  N  P  K  L  K  T  I   100
 301 gccggtgttggaggatggaatgaaggaagtgttacattttctcaggtcgtcagtgatcca
      A  G  V  G  G  W  N  E  G  S  V  T  F  S  Q  V  V  S  D  P   120
 361 cgcaagaggcagaatttcgtgaagaattcgttggaattttgaaaaaatacaattttaat
      R  K  R  Q  N  F  V  K  N  S  L  E  F  L  K  K  Y  N  F  N   140
 421 ggtcttgacgttgactgggagtatccagcacaacgtggagggaatcaggataaggataaa
      G  L  D  V  D  W  E  Y  P  A  Q  R  G  G  N  Q  D  K  D  K   160
 481 gaagcctacacgctccttctcaaggaactctcggaattcctacatcctaaaggctacact
      E  A  Y  T  L  L  L  K  E  L  S  E  F  L  H  P  K  G  Y  T   180
 541 ctctccgctgctgtagcatcagcggaattttcagctaaaatctcttacaacatcgctgag
      L  S  A  A  V  A  S  A  E  F  S  A  K  I  S  Y  N  I  A  E   200
 601 gtttcaaaatatctggatttcattggggtaatgacgtatgatttacacggatcatgggat
      V  S  K  Y  L  D  F  I  G  V  M  T  Y  D  L  H  G  S  W  D   220
 661 ccaaagattgggaataatgcgcctttgtatgcgggatcgtgggagcaaacggagcttgag
      P  K  I  G  N  N  A  P  L  Y  A  G  S  W  E  Q  T  E  L  E   240
 721 aagcaattaaatgttgacgctgctataaagtactggctcagcaatagcggggctcctgag
      K  Q  L  N  V  D  A  A  I  K  Y  W  L  S  N  S  G  A  P  E   260
 781 aagctcctcctgggcgttccactgtacggtcgtggcttccgcatggttaatggtcagagt
      K  L  L  L  G  V  P  L  Y  G  R  G  F  R  M  V  N  G  Q  S   280
 841 aaaccaggatccgttcatggaggtccttgtcaggctgggccctacactcaaactcccggc
      K  P  G  S  V  H  G  G  P  C  Q  A  G  P  Y  T  Q  T  P  G   300
 901 atgatgggcttcaatgagctctgcgagaagcgtcagaatgaaaaatggattgatttctgg
      M  M  G  F  N  E  L  C  E  K  R  Q  N  E  K  W  I  D  F  W   320
 961 gatgatgagcaatttgttccctactccacaaagaacgatcagtggattggatttgatgat
      D  D  E  Q  F  V  P  Y  S  T  K  N  D  Q  W  I  G  F  D  D   340
1021 gagaagagtataaaattcaaatcaaattacgtcaacagccacaatcttggtggtgtgatt
      E  K  S  I  K  F  K  S  N  Y  V  N  S  H  N  L  G  G  V  I   360
1081 gtgtggtccattgaaacagatgattttagaggattctgcggaagaggaaccttcccactt
      V  W  S  I  E  T  D  D  F  R  G  F  C  G  R  G  T  F  P  L   380
1141 ctcaaggagctcaatgcatccctccttggtaacaatgtgagtacctccaatgaaacagac
      L  K  E  L  N  A  S  L  L  G  N  N  V  S  T  S  N  E  T  D   400
1201 cccgagatcatgtccctcctttccgcggagaagaagaaaacagcagcattgaagaaattc
      P  E  I  M  S  L  L  S  A  E  K  K  K  T  A  A  L  K  K  F   420
1261 attcatgatttcttcttcaagaccttccagccaaagtatcagaaactcattcagttcaat
      I  H  D  F  F  F  K  T  F  Q  P  K  Y  Q  K  L  I  Q  F  N   440
1321 ttactcaaagaaaaatatttttctgaaagcttcaccgaactctacggtacatacgttcaa
      L  L  K  E  K  Y  F  S  E  S  F  T  E  L  Y  G  T  Y  V  Q   460
1381 atgctctcagacacatacaagagtgaaaatgatatataagcaggaattaagtttatttc
      M  L  S  D  T  Y  K  S  E  N  D  I  -                        472
1441 tcttaattttgtgtttttttttttattgagaaataataagtatttaatgcaatttacgga
1501 aaataaaaccactttgcgcggatttcaagtgatttagcacttgagggcattcttcattgg
1561 ctgagagggtgcgagctttgttttctgctgcacttccggatatctacgaaagcaggaaga
1621 cacacctggacacctccatcaacatcaaccaccgaatggaatcccaatccaacgtccaca
1681 acgaaaccaccaccaatcctcccaagtcctgaatgtactgaagatggttactttagggat
1741 ccaaatgactgcagtatcttccaccaatgcatcaatgggactcaatatacattcttctgc
1801 ccacatggtcttgtcttcgatccagccatcatagcttgcaattggccccatatagtgcaa
1861 tgctga
```

Fig. 5: LlChit1C spliced form sequence: numbers on left: nucleotide position. Numbers on right: amino acid position. Asterisk: putative signal peptide cleavage site. Red letters: conserved catalytic domain. Red underlined bold letters: catalytic core.

TABLE I

Sequences used in phylogenetic tree analysis

Drosophila melanogaster

	Domain 1			Domain 2			Domain 3			Domain 4		
Gene name	start	end	length	start	end	length	start	end	length	start	end	length
DmCht2	41	389	349									
DmCht4	24	351	328									
DmCht5	27	379	353									
DmCht6	52	405	354									
DmCht7	123	468	346	558	898	341						
DmCht8	13	363	351									
DmCht9	21	346	326									
DmCht10	219	565	347	965	1310	346	1409	1753	345	1908	2253	346
DmCht11	67	398	332									
DmCht12	23	355	333									
DmIDGF1	22	417	396									
DmIDGF2	22	419	398									
DmIDGF3	25	419	395									
DmIDGF4	25	420	396									
DmIDGF5	29	423	395									
DmIDGF6	29	429	401									

Aedes aegypti

	Domain 1			Domain 2			Domain 3			Domain 4		
Accession number	start	end	length	start	end	length	start	end	length	start	end	length
XP_001657537.1	10	346	337									
XP_001656234.1	33	385	353									
XP_001656233.1	30	331	302									
XP_001656232.1	35	379	345									
XP_001656231.1	35	379	345									
XP_001662588.1	43	396	354									
XP_001650020.1	122	467	346	555	895	341						
XP_001663097.1	24	371	348									
XP_001656054.1	10	352	343	439	780	342						
XP_001655973.1	201	548	348	1005	1350	346	1432	1777	346	2025	2370	346
XP_001654045.1	126	465	340									
XP_001663568.1	28	366	339									
XP_001655071.1	26	361	336									
XP_001663099.1	24	371	348									
XP_001660745.1	25	420	396									
XP_001660748.1	28	420	393									

TABLE II

Primers

Number	Name	Sequence
1	EMBL3-F	CGTGAAAGGTAGGCGG
2	Chit G-R	AAGGCGACACACAGAAACAC
3	Chit N-F	ATGAAGACGTTGGTGTTTCTG
4	Chit int 6-R	ATCCGTGTAAATCGTA
5	Chit int 5-F	GCAGTGGCATCAGCGG
6	Chit RNA 2-R	CTCGAGCATGATCCGTGTAAAT
7	Chit His-F	ACAAAGAACGATCAGT
8	Chit1.2-R	GATCGTTCTTTGTGGAGT
9	RT Chit1-F	TTGTGTGGTCCATTGAAACAGATGA
10	Chit int 3-R	TTAGGGGATTTAGCGA
11	Chit int 2-F	CTACGAAAGCAGGAAG
12	Chit His-R	TCAGCATTGCACTATATGGGGCCA
